# Circulating sphingosine-1-phosphate and erythrocyte sphingosine kinase-1 activity as novel biomarkers for early prostate cancer detection

**DOI:** 10.1038/bjc.2012.14

**Published:** 2012-02-07

**Authors:** J Nunes, M Naymark, L Sauer, A Muhammad, H Keun, J Sturge, J Stebbing, J Waxman, D Pchejetski

**Affiliations:** 1 Department of Surgery and Cancer, Imperial College London, Cyclotron Building, Ducane Road, London W12 0NN, UK; 2 Department of Biomolecular Medicine, Imperial College London, Alexander Fleming Building, Imperial College Road, London SW7 2AZ, UK

**Keywords:** prostate cancer, molecular marker, diagnosis, prognosis, sphingosine-1-phosphate, anaemia

## Abstract

**Background:**

Current markers available for screening normal populations and for monitoring prostate cancer (PCa) treatment lack sensitivity and selectivity. Sphingosine-1-phosphate (S1P) is a circulating lipid second messenger involved in cell growth and migration, the immune response, angiogenesis, and malignant transformation.

**Methods:**

Eighty-eight patients with localised, locally advanced, or metastatic PCa were recruited into this prospective single-centre study. Plasma S1P levels were measured and compared with age-matched controls with benign prostate hyperplasia (BPH) (*n*=110) or with young healthy males with the very small chance of having PCa foci (*n*=20).

**Results:**

Levels of circulating S1P were significantly higher in healthy subjects (10.36±0.69 pmol per mg protein, *P*<0.0001) and patients with BPH (9.39±0.75, *P*=0.0013) than in patients with PCa (6.89±0.58, ANOVA, *P*=0.0019). Circulating S1P levels were an early marker of PCa progression to hormonal unresponsiveness and correlated with prostate-specific antigen (PSA) levels and lymph node metastasis. During the course of the study, nine patients have died of PCa. Importantly, their circulating S1P levels were significantly lower (5.11±0.75) than in the surviving patients (7.02±0.22, *n*=79, *P*=0.0439). Our data suggest that the decrease in circulating S1P during PCa progression may stem from a highly significant downregulation of erythrocyte sphingosine kinase-1 (SphK1) activity (2.14±0.17 pmol per mg protein per minute in PCa patients *vs* 4.7±0.42 in healthy individuals, *P*<0.0001), which may be a potential mechanism of cancer-induced anaemia.

**Conclusion:**

This current study has provided a potential mechanism for cancer-related anaemia and the first evidence that plasma S1P and erythrocyte SphK1 activity are the potential markers for the diagnosis, monitoring, and predicating for PCa mortality.

In the Western World, prostate cancer (PCa) is now the most commonly diagnosed non-cutaneous cancer in men and is the second leading cause of cancer-related death (ACS, 2011). In the United States, the lifetime probability of developing PCa is one in six and it is estimated that 240 890 new cases of PCa will be diagnosed in 2011, and there will be 33 720 deaths.

The only available blood test for PCa involves measuring circulating levels of prostate-specific antigen (PSA), a protease secreted by prostate cells. However, the prostate size, the presence of benign prostate hyperplasia (BPH), and prostatitis are all known to increase plasma levels of PSA resulting in only 33% of patients above the currently accepted normal assay limit of 4.0 ng ml^−1^ having PCa. Conversely, up to 26% of patients with PSA <4.0 ng ml^−1^ will have PCa (Thompson *et al*, 2004). In advanced PCa, oncologists have mainly focused on PSA kinetics as a clinical surrogate for outcome. However, although changes in PSA levels do give a general prognosis they lack specificity for the individual (Nelius and Filleur, 2009).

Sphingosine-1-phosphate is a lipid second messenger that mediates processes with important roles in cancer progression, angiogenesis, and cell motility, making it a key molecule in the search for potential anticancer therapies. It is secreted into the extracellular milieu and binds to specific transmembrane G protein-coupled S1P receptors (S1PRs) to promote cell growth, migration, chemotaxis, and inflammatory response.

Sphingosine kinase-1 (SphK1), the enzyme that generates S1P has oncogenic properties (Xia *et al*, 2000), contributes to cancer progression (Olivera *et al*, 1999), enhances tumour neovascularisation (Licht *et al*, 2003) and metastatic potential (Visentin *et al*, 2006) and is associated with a poor prognosis (Ruckhaberle *et al*, 2008). Recent work in our laboratory has demonstrated the role of the SphK1/S1P pathway in PCa progression, disease recurrence, and invasion into the extracapsular space (Malavaud *et al*, 2010). *In-vitro* and *in-vivo* studies indicate a significant translational potential for the SphK1-targeting therapies (Pchejetski *et al*, 2005, 2008, 2010a; Sauer *et al*, 2009). Recent data show that FTY720 (Fingolimod), an agonist of S1PRs 1, 3, 4, and 5, which is clinically used as a functional S1PR antagonist in treatment of multiple sclerosis is the first clinically applicable SphK1 inhibitor and has a radiosensitising potential providing improved local control for prostate tumours (Pchejetski *et al*, 2010a).

Sphingosine-1-phosphate is present in the circulation in high nanomolar concentrations, and erythrocytes (Hanel *et al*, 2007; Ohkawa *et al*, 2008) are considered to be its major source. The potential of S1P to have a role as a plasma marker of human disease has been recently demonstrated by a study, which showed that plasma S1P was elevated in patients with inflammatory coronary artery disease (Deutschman *et al*, 2003). Circulating levels of S1P were shown to be higher in mice with colon cancer (Kawamori *et al*, 2006) and diabetes (Fox *et al*, 2011). In human ovarian cancer patients, S1P levels were higher than in controls, although they were not correlated with clinical stage (Sutphen *et al*, 2004). We have recently associated a modulation of circulating S1P with aromatase inhibitor-induced arthralgia (Henry *et al*, 2010) and chemotherapy-induced weight gain (Pchejetski *et al*, 2010b).

In this current report, we show that the levels of circulating S1P in healthy individuals and patients with BPH are significantly higher as compared with PCa. Furthermore, there is a significant correlation between plasma S1P and clinical correlates in PCa (PSA, PCa progression). Finally, our data provide the first evidence that circulating S1P is a significant prognostic marker for PCa mortality. We have identified erythrocytes as the major source of S1P in our patients and shown a highly significant downregulation of erythrocyte SphK1 activity in PCa patients compared with control counterparts, suggesting a mechanism for PCa-induced anaemia.

Overall, in this study we provide the first evidence that both plasma S1P and erythrocyte SphK1 activity have potential as diagnostic and prognostic markers for human PCa.

## Materials and methods

### Subjects and study design

In all, 116 PCa patients attending the Oncology Clinic, Hammersmith Hospital (London, UK) were approached and 88 PCa patients were recruited into this prospective single-centre study from February 2005 through October 2005 and their progress tracked until October 2008 (Hammersmith hospitals Research Ethics Committee approval 2000/5816). Sample size: an *a priori* power analysis (SPSS software, SPSS Inc., Chicago, IL, USA) indicated that *n*=24 patients per group (e.g., disease stage or treatment, independent samples) would be required to detect correlation between variables (*α*=5% probability type 1 error, significance level; 95% power; 50% confidence interval, and 40% standard deviation). To ensure a comprehensive analysis of various types of PCa patients that attend a typical oncology clinic, all eligible patients were approached (no stratification). Inclusion criteria (CONSORT table in Supplementary Figure S1 and Supplementary Table S1) was PSA levels <1000, absence of multiple metastases. Control groups included 110 age-matched patients with BPH, several patients with other cancers (Supplementary Figure S2) as well as healthy volunteers (*n*=20). At the time of recruitment into the study (time=0), TNM staging and Gleason score assessment were performed, and 20 ml of blood was taken for blood counts, PSA and testosterone analysis, and serum S1P measurement. CT scan, MRI, and biopsies followed by staging were performed on all patients at initial staging. Patients were then assigned a standard treatment according to their clinical assessment (CONSORT table in Supplementary Figure S1). Patients were re-evaluated 6 and 36 months after recruitment with PSA re-measured. At these time points, S1P measurements were not taken due to the limitation of the ethical approval to one intervention. At the end of study, clinical end points (survival, stage, Gleason score, metastasis, PSA, and testosterone) were re-assessed.

### Reagents

[*γ*-^32^P]-ATP (6000 mCi mmol^−1^) was purchased from Perkin-Elmer (Waltham, MA, USA) and silica gel 60 high-performance TLC plates were from GE Healthcare (Waukesha, WI, USA). All other chemicals and reagents were obtained from Sigma-Aldrich (Carlsbad, CA, USA).

### Plasma S1P analysis

Samples were collected in heparin tubes, put on ice, transferred to the laboratory and plasma was separated by centrifugation, aliquoted, and frozen. Plasma S1P content was measured similarly to a method previously described by Edsall and Spiegel (1999). Briefly, 50 *μ*l of serum was used for chloroform/methanol lipid extraction in alkaline conditions. To aqueous phase buffer C (200 mmol L^−1^ Tris–HCl (pH 7.4), 75 mmol L^−1^ MgCl_2_ in 2 mol L^−1^ glycine (pH 9.0)) was added (1 : 6, v/v) and S1P was dephosphorylated by addition of 50 units per sample of alkaline phosphatase for 30 min at 37 °C. Reaction was stopped by addition of HCl and organic phase containing sphingosine was separated and evaporated. Evaporated sphingosine was re-suspended in sphingosine kinase buffer with 0.25% Triton X-100 and converted to S1P by addition of recombinant bacterial SphK1 and [*γ*-32P]ATP (10 *μ*Ci, 1 mmol L^−1^) containing 10 mmol L^−1^ MgCl_2_. Sphingosine-1-phosphate was resolved by TLC, measured by autoradiography and expressed as picomoles of S1P formed per mg of plasma protein.

### Blood cell isolation and culture

Fresh blood samples from control subjects and PCa patients were collected in heparin tubes. Plasma was separated by centrifugation; peripheral blood mononuclear cells (PBMCs) and erythrocytes were isolated by centrifugation on Ficoll-Hypaque gradients; snap frozen in liquid nitrogen and kept at −80 °C or cultured in the RPMI-1640 media with 10% FBS in the presence or absence of 30% fresh plasma, fibroblasts, or PCa cells.

### Cell lines

Human PCa PC-3 and DU145 cells were obtained from DSMZ (Braunschweig, Germany) and the immortalised human dermal fibroblasts fibroblast cell line HCA was obtained from Prof David Kipling (Cardiff University). All cells were cultured between passages 4 and 30 in RPMI-1640 containing 10% FBS. Cell lines were routinely verified by morphology and growth curve analysis and routinely screened for mycoplasma infection (MP0035 Lookout (R) mycoplasma PCR kit; Sigma-Aldrich). Cancer cell/erythrocyte or fibroblast/erythrocyte co-culture experiments were conducted in transwell inserts, in the presence of serum at 50% confluence (for adherent cells).

### SphK1 assay

The SphK1 assay was performed as previously described (Bonhoure *et al*, 2006). Briefly, upon treatment, red blood cells (RBCs) were harvested by centrifugation, washed with ice-cold PBS, and cell pellets were re-suspended in SPHK buffer (20 mM Tris (pH 7.4)), 20% glycerol, 1 mM *β*-mercaptoethanol, 1 mM EDTA, 1 mM sodium orthovanadate, 40 mM *β*-glycerophosphate, 15 mM NaF, 1 mM phenylmethylsulphonyl fluoride, 10 *μ*g ml^−1^ leupeptin, 10 *μ*g ml^−1^ aprotinin, 10 *μ*g/ml soybean trypsin inhibitor, and 0.5 mM 4-deoxypyridoxine. After 10 s sonication, samples were ultracentrifuged for 90 min (105 000 **g** at 4 °C). The SphK1 activity was determined in the cytosolic fractions in the presence of 50 *μ*M sphingosine (Avanti Polar Lipids, Alabaster, AL, USA), 0.25% Triton X-100 and 20 mM ATP containing 10 *μ*Ci [*γ*-32P]-ATP and 10 mM MgCl_2_. The labelled S1P was separated by thin layer chromatography on silica gel G60 with 1-butanol/ethanol/acetic acid/water (80 : 20 : 10 : 10, v/v) and quantified by autoradiography (ImageJ software, US NIH, Bethesda, MD, USA). The SphK1-specific activity was expressed as picomoles of S1P formed per min per mg of protein.

### Determination of blood PSA and testosterone

PSA and testosterone serum levels were tested using AxSYM PSA assay and AxSYM testosterone assay, respectively (Abbott Laboratories, Maidenhead, UK).

### Data representation and statistical analysis

Data analysis and presentation were performed in accordance with CONSORT and REMARK recommendations and did not include any multiple testing, variable selection, and cut point optimisation. All measurements were performed in blinded manner. The statistical significance of differences between the means of two groups was evaluated by the non-parametric Mann–Whitney *U*-test for categorical data, and the unpaired two-tailed *t*-test was used for continuous data. When three or more groups were analysed, one-way ANOVA test has been used. Correlation analyses were performed using Pearson's correlation test.

## Results

### Plasma S1P levels decrease during early PCa progression and correlate with TN status

**Figure 1:**
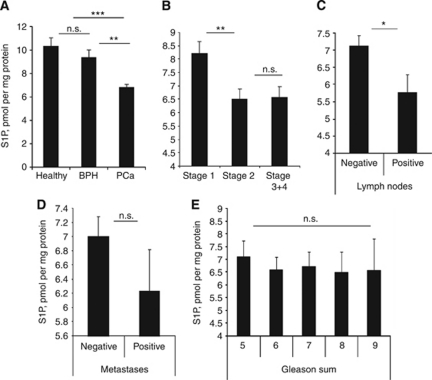
Circulating S1P levels correlate with PCa onset and TN status. At the time of patients’ recruitment, a TNM and Gleason score assessment was performed, and 20 ml of blood was taken for blood counts, PSA and testosterone analysis, and plasma S1P measurement. Plasma S1P levels in healthy individuals, BPH, and PCa patients (**A**); patients with PCa stage 1, 2, and 3+4 (**B**); PCa patients with negative and positive lymph nodes (**C**); PCa patients with or without metastases (**D**); PCa patients with different Gleason sum (**E**). Columns, mean values; Bars, s.e.m.; n.s., not significant; ^*^P<0.05; ^**^P<0.001; ^***^P<0.0001.

Circulating levels of S1P were measured in plasma samples of BPH (*n*=110) and PCa (*n*=88) patients or control patients with no history of cancer (*n*=20), and correlated with the clinical stage and grade. Levels of circulating S1P were significantly higher in healthy subjects (10.36±0.69 pmol per mg protein, *P*<0.0001) and patients with BPH (9.39±0.75, *P*=0.0013) than in patients with PCa (6.89±0.58) (ANOVA, *P*=0.0019; Figure 1A). Importantly, within the PCa group plasma S1P inversely correlated with the stage of the disease (Figure 1B, ANOVA, *P*=0.0456) with T1 patients having significantly higher levels of circulating S1P than T2–T4 patients (T1: 8.21±0.44 *vs* T2: 6.52±0.36, *P*=0.0085; T3+4: 6.57±0.41 pmol per mg protein).

**Figure 2:**
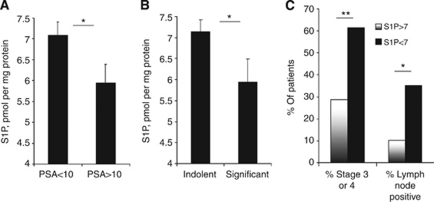
Low levels of circulating S1P in PCa patients correlate with the plasma levels of PSA and testosterone and are a marker of significant disease. Plasma S1P levels in PCa patients with lower (<10) and higher (>10) PSA (**A**); indolent (stage 1 or stage 2 PCa, Gleason score 3+3) and significant (stage 3 or stage 4 metastatic PCa, Gleason score of 4+4 or greater) disease (**B**). Percent of high stage tumours and positive lymph nodes in PCa patients with lower (<7) and higher (>7) S1P (**C**). Columns, mean values; Bars, s.e.m.; ^*^P<0.05; ^**^P<0.001.

Decrease in plasma S1P has also significantly correlated with positive lymph nodes (Figure 1C, 7.12±0.29 (negative, *n*=73) *vs* 5.77±0.51 (positive, *n*=15), *P*=0.0308). Conversely, plasma S1P was neither correlated with the presence of PCa metastasis (Figure 1D, *P*=0.2017), nor with Gleason sum (Figure 1E, ANOVA, *P*=0.9940). Plasma levels of S1P were only slightly higher in patients with Gleason sum 5 in comparison patients with Gleason sum 6 (7.09±0.65 *vs* 6.58±0.51, respectively, *P*=0.6512). Plasma levels of S1P were inversely correlated with plasma PSA levels (Figure 2A). In patients with PSA>10 ng ml^−1^, S1P levels were significantly lower than in patients with the PSA<10 ng ml^−1^ (5.95±0.44 *vs* 7.08±0.32, respectively, *P*=0.046). Similarly, patients with S1P<7 pmol per mg protein had ∼three-fold higher PSA levels than patients with S1P>7 pmol per mg protein (105.3±56.3 *vs* 37.28±30.06, ns; due to significant variation in PSA levels between patients).

We have then grouped patients into those men with a relatively indolent PCa (stage 1 or stage 2 PCa, and an average Gleason score 3+3) and compared them with patients with PCa of more profound clinical significance defining this group as having either stage 3 or stage 4 PCa, having high metastatic occurrence and a Gleason score of 4+4 or greater. As shown in Figure 2B, patients with indolent PCa had significantly higher S1P than patients with clinically more aggressive disease (7.14±0.28 *vs* 5.93±0.55, respectively, *P*=0.0406).

We have then divided the patients on the groups with low (<7 pmol per mg protein) and high (>7 pmol per mg protein) S1P. The ‘low S1P’ group contained a statistically greater proportion of patients with stage 3 or 4 tumours (*P*=0.0025) and positive lymph nodes (*P*=0.015) than the ‘high S1P’ group (Figure 2C).

### S1P correlates with plasma PSA, but is an earlier marker of PCa

**Figure 3:**
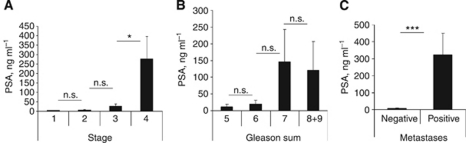
PSA is a marker of late PCa. Plasma PSA levels in PCa patients: with different stages of disease (**A**); with different Gleason sum (**B**); with or without metastases (**C**). Columns, mean values; Bars, s.e.m.; n.s., not significant; ^*^P<0.05; ^***^P<0.0001.

Circulating S1P correlates with plasma PSA (Figure 2A). Figure 3A shows that PSA levels significantly correlated with tumour progression (ANOVA, *P*=0.0027); however, in contrast to S1P, PSA was rather a late than an early marker gaining significance only during progression from stage 3 to stage 4 (27.15±12.67 *vs* 278.50±117.92, respectively, *P*=0.0463). Prostate-specific antigen was not a significant predictor of Gleason sum (Figure 3B, ANOVA, *P*=0.4282), but was however significantly elevated in patients with metastatic PCa (Figure 3C, 8.73±2.00 (negative) *vs* 323.20±127.8 (positive), *P*<0.0001).

It is well known that cancer treatments affect plasma testosterone levels (Iversen *et al*, 1994), we have therefore correlated plasma testosterone with S1P and clinical symptoms only in patients that received no prior treatment (*n*=39). Unlike the PSA, circulating S1P levels did not correlate with plasma testosterone levels (data not shown).

**Figure 4:**
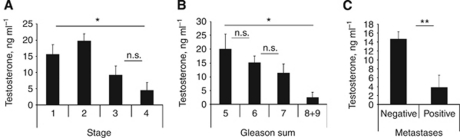
Testosterone is a marker of late PCa. Plasma testosterone levels in untreated PCa patients: with different stages of disease (**A**); with different Gleason sum (**B**); with or without metastases (**C**). Columns, mean values; Bars, s.e.m.; n.s., not significant; ^*^P<0.05; ^**^P<0.001.

Plasma testosterone levels significantly correlated with PCa stage (Figure 4A, ANOVA, *P*=0.002); however similarly to PSA, plasma testosterone levels in T1 and T2 patients were undistinguishable. In contrast to PSA, plasma testosterone was significantly correlated with Gleason sum (Figure 4B, ANOVA, *P*=0.0495) and with the appearance of metastases (Figure 4C, 14.66±1.65 (negative) *vs* 3.770±2.78 (positive), *P*=0.0047).

### Plasma S1P has a prognostic value in human PCa

**Figure 5:**
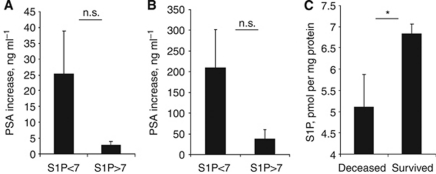
Correlation of circulating S1P levels with PSA progression and patient survival. Six months plasma PSA increase in PCa patients with low (<7) and high (>7) plasma S1P (**A**); 36 months plasma PSA increase in PCa patients with low (<7) and high (>7) plasma S1P (**B**). (**C**) Plasma S1P levels (time=0) in deceased *vs* survived PCa patients. Columns, mean values; Bars, s.e.m.; n.s., not significant; ^*^P<0.05.

Prostate-specific antigen changes following the diagnosis of malignancy are used to mark disease progression. We have quantified 6 and 36 months PSA change in individual patients and correlated these levels with time=0 plasma S1P levels. Our data show no significant correlation between PSA increase and circulating S1P. However, although not significantly different, patients with lower plasma S1P levels (<7) had nine-fold higher average 6 months PSA increases than patients with higher S1P levels (>7) (Figure 5A, 25.30±13.66 *vs* 2.77±1.23, respectively, *P*=0.1543). A very similar pattern was observed after 36 months – the PSA increase was ∼5.6-fold higher in patients with lower pre-entry S1P levels (Figure 5B, 209.1±92.95 *vs* 37.36±22.79, *P*=0.098).

At the point of analysis, 9 patients have died of PCa and 79 patients were alive. Circulating S1P levels (at time 0) in the deceased were 5.11±0.75 *vs* 6.84±0.22 pmol per mg protein in the surviving patients (*P*=0.0439), allowing to propose circulating S1P as a significant prognostic marker predicating for PCa mortality (Figure 5C).

### The reduction of circulating S1P in PCa patients correlates with a decrease in erythrocyte SphK1 activity

Erythrocytes (Hanel *et al*, 2007; Ohkawa *et al*, 2008) have been suggested as being the major source of circulating S1P. In PCa patients, Pearson's correlation testing showed a significant correlation between S1P and white blood cells (WBCs) counts (Supplementary Table S2), but not between S1P and , platelets or haemoglobin. However, neither blood cell counts nor HgB statistically correlated with stage, Gleason sum, and PSA levels (Supplementary Table S2).

**Figure 6:**
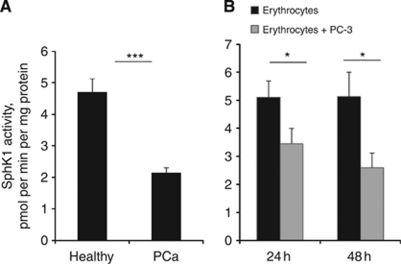
RBC SphK1 activity is a marker of PCa presence. SphK1 activity in RBCs (**A**) isolated from healthy individuals or PCa patients. (**B**) SphK1 activity in RBCs isolated from healthy controls cultured in the presence or absence of prostate cancer cells (PC-3) for indicated time as described in Materials and methods. Columns, mean values; Bars, s.e.m.; ^*^P<0.01; ^***^P<0.0001.

We have then isolated RBCs from healthy controls and individuals with PCa and compared their SphK1 activity (normalised per cellular protein content). Erythrocyte SphK1 activity in PCa patients was >2-fold lower than in healthy controls (2.14±0.17 pmol per mg protein per minute in PCa *vs* 4.71±0.42 in healthy individuals, *P*<0.0001) (Figure 6A). Importantly, there were no overlaps between these groups, suggesting that erythrocyte SphK1 activity is a potential biomarker for PCa diagnosis. This significant change of SphK1 activity was not reflected by lower RBC counts in both PCa patients and healthy controls (Supplementary Table S3), indicating that cancer presence may modify erythrocyte cell SphK1 activity irrespective of erythrocyte numbers. Of note, platelet and WBC counts and intracellular SphK1 activity in PCa patients did not statistically differ from BPH patients and healthy controls (data not shown).

We have then correlated RBCs SphK1 activity with circulating levels of S1P. As shown in Supplementary Table S3, in both healthy volunteers and PCa patients SphK1 activity in RBCs was correlated with circulating S1P (*P*=0.031 and *P*=0.043, respectively). Interestingly, co-culturing (using transwell inserts) of PC-3 PCa cells with erythrocytes isolated from healthy controls has induced a decline in erythrocyte SphK1 activity (Figure 6B). Similar data were obtained with DU145 PCa cells, but not with human fibroblasts (Supplementary Figure S3). These data indicated that PCa cells may potentially secrete a factor that is downregulating erythrocyte SPHK1 activity. Further experiments demonstrated that similarly to incubation with PCa cells fresh plasma from PCa patients’ inhibited SphK1 activity in RBCs derived from healthy controls (Supplementary Figure S4). On the contrary, incubation with plasma from healthy controls did not decrease SphK1 activity in RBCs derived from PCa patients, which showed a tendency to regain SphK1 activity (Supplementary Figure S4).

### In PCa patients, circulating levels of S1P are unaffected by age or treatment regimen

Circulating S1P has neither correlated with age (Supplementary Figure S2A) nor with chemotherapy regimen (Supplementary Table S4, ANOVA, *P*=0.079). Comparison of S1P values in any of the two groups of patients did not show a statistically significant difference. Furthermore, therapy-induced modifications of blood cell counts showed no correlation with changes in plasma S1P (data not shown, in all cases Pearson's *P*-value was >0.1).

In addition to PCa patients, we have analysed plasma S1P in patients with various cancers (Supplementary Figure S2B). Although the number of recruited patients was insufficient to provide any statistically significant data, our results show that patients with each type of cancer have distinct levels of plasma S1P with breast cancer patients having the lowest plasma S1P (5.02±0.80) and patients with teratoma having the highest plasma S1P (9.24±2.91), suggesting that this may reflect the way different tumours induce paraneoplastic phenomenon.

## Discussion

In this current study, we provide compelling evidence that circulating S1P and erythrocyte SphK1 activity are potential diagnostic and prognostic markers for human PCa.

Our results indicate that plasma S1P levels were lower in patients with PCa than in men with benign prostatic hypertrophy or healthy controls and correlated with disease state (Figure 1). Plasma S1P was also significantly lower in patients with positive lymph nodes, but did not correlate with the presence of distant metastases or Gleason score. These data suggest that the observed drop in the circulating S1P is correlated with an early cancer progression as opposed to acquiring of clinically metastatic status. In line with this hypothesis, circulating S1P had a prognostic value in distinguishing between those patients with indolent as compared with clinically significant PCa (Figure 2C).

Plasma S1P was significantly correlated with plasma levels of PSA (Figure 2), but not testosterone. In contrast to S1P, both PSA (Figure 3) and testosterone (Figure 4) appeared to be rather late predictors of PCa progression – no significant differences were observed between patients with early stages of PCa. Conversely, both markers were highly correlated with metastatic status of PCa patients (Lin, 2009).

Longitudinal analysis (Figure 5) showed that patients with low plasma S1P (<7 pmol per mg protein, *n*=67) had 9-fold and 5.6-fold higher average 6 and 36 months PSA increase than patients with high S1P (>7 pmol per mg protein, *n*=21). While S1P correlation with PSA increase was not statistically significant (due to a considerable difference in PSA levels in different patients), plasma S1P was significantly correlated with PCa patient mortality (Figure 5C). These data clearly demonstrate that plasma S1P might not only be an early marker of the PCa onset (Figure 1), but may serve as a prognostic factor of the PCa outcome (Figure 5). In isolated human samples, S1P shows significant stability. While in live organism S1P has a very quick turnover (half-life ∼15 min; Venkataraman *et al*, 2008), prolonged storing of blood samples or plasma at 4 °C did not alter S1P concentration (data not shown).

The fact that circulating levels of S1P were lower in patients with advanced PCa (Figure 1) ruled out the possibility of circulating S1P to originate from cancer cells. Sphingosine-1-phosphate is present in high concentrations in circulating haematopoietic cells, and in our patients there was a positive correlation between plasma S1P and WBC counts (Supplementary Table S2), although surprisingly we have found no correlation with platelet or red cell numbers or haemoglobin levels (Supplementary Table S2). We have shown that blood cell counts *per se* were not relevant to PCa stage, Gleason sum, and PSA levels (Supplementary Table S5). Dividing PCa patients according to their age or therapies revealed that S1P has neither correlated with age (Supplementary Figure S2) nor with chemotherapy regimen (Supplementary Table S4), suggesting that in these patients circulating S1P levels are determined rather by cancer presence than by these factors.

We hypothesised that the observed changes in plasma S1P are most probably a paraneoplastic phenomenon affecting S1P production and secretion by blood cells. This hypothesis was further confirmed by the fact that plasma S1P levels differed between patients with different cancers (Supplementary Figure S2B).

Anaemia is a common problem in PCa patients, many of whom are anaemic at presentation and experience its clinical manifestations (e.g., fatigue and dyspnoea). However, the exact mechanism of cancer-induced anaemia is currently not known. Our data provide the first evidence that cancer presence may significantly downregulate erythrocyte SphK1 activity (Figure 6A) and this significantly correlates with circulating S1P both in healthy individuals and in PCa patients (Supplementary Table S3). This coincides with a recent report showing that an SphK1 inhibitor FTY720 can induce RBCs cell death (Eberhard *et al*, 2010). Our data indicate that PCa cells may potentially secrete a factor that decreases RBC SphK1 activity and this factor is present in the plasma of PCa patients (Supplementary Figures 6B, S3, S4, and S5).

Lower plasma levels of S1P may lead to several additional side effects that may influence the course of the disease and the efficacy of anticancer therapies. Sphingosine-1-phosphate was demonstrated to have a key role in vascular permeability (Garcia *et al*, 2001) and recently low levels of circulating S1P were linked with progression of leaky vessels (Camerer *et al*, 2009), a known phenomenon in tumour biology that impairs chemotherapy access to tumour cells. Furthermore, plasma S1P has been shown to have a protective role on the cardiovascular system (Rodriguez *et al*, 2009), which may be particularly important in a view of a recent report of increased relative risks of cardiovascular disease in all men with PCa (Van Hemelrijck *et al*, 2010).

Overall, in this study we have identified plasma S1P as a new diagnostic and prognostic marker for PCa. Importantly, the biggest change in plasma S1P was detected in patients with the early stage of PCa, who were not yet treated with any therapy. This suggests that plasma S1P may be used as a potential biomarker for early PCa, especially in a view that high throughput methods of its analysis are available (Bielawski *et al*, 2006). While our data indicate that in PCa patients circulating levels of S1P are unaffected by age or treatment regimen, due to limited group sizes the links between S1P and therapy should be investigated further. In this study, regression and multivariate analyses were not used as the small sample size would have limited statistical power for these analyses. More studies in stratified groups should be performed to identify the diagnostic/prognostic potential of plasma S1P in various patients groups and in patients with other cancers. We have found that plasma S1P is secreted from RBCs and is decreased during the early PCa progression, which reflects a functional change in the RBCs SphK1 activity and not RBC counts in response to cancer presence. These data suggest that circulating S1P levels may reflect early metabolic changes in PCa patients and serve as independent predictors, specifically in younger patients with aggressive tumours and low PSA counts. Further studies are required to investigate the exact mechanism of paraneoplastic changes in blood cell SphK1 activity and its implication in cancer progression.
