# Supplementary figures and images for: Circulating sphingosine-1-phosphate and erythrocyte sphingosine kinase-1 activity as novel biomarkers for early prostate cancer detection
Source: Br J Cancer. 2012 Feb 7;106(5):909–15. doi: 10.1038/bjc.2012.14 (PMC3305969; doi:10.1038/bjc.2012.14)

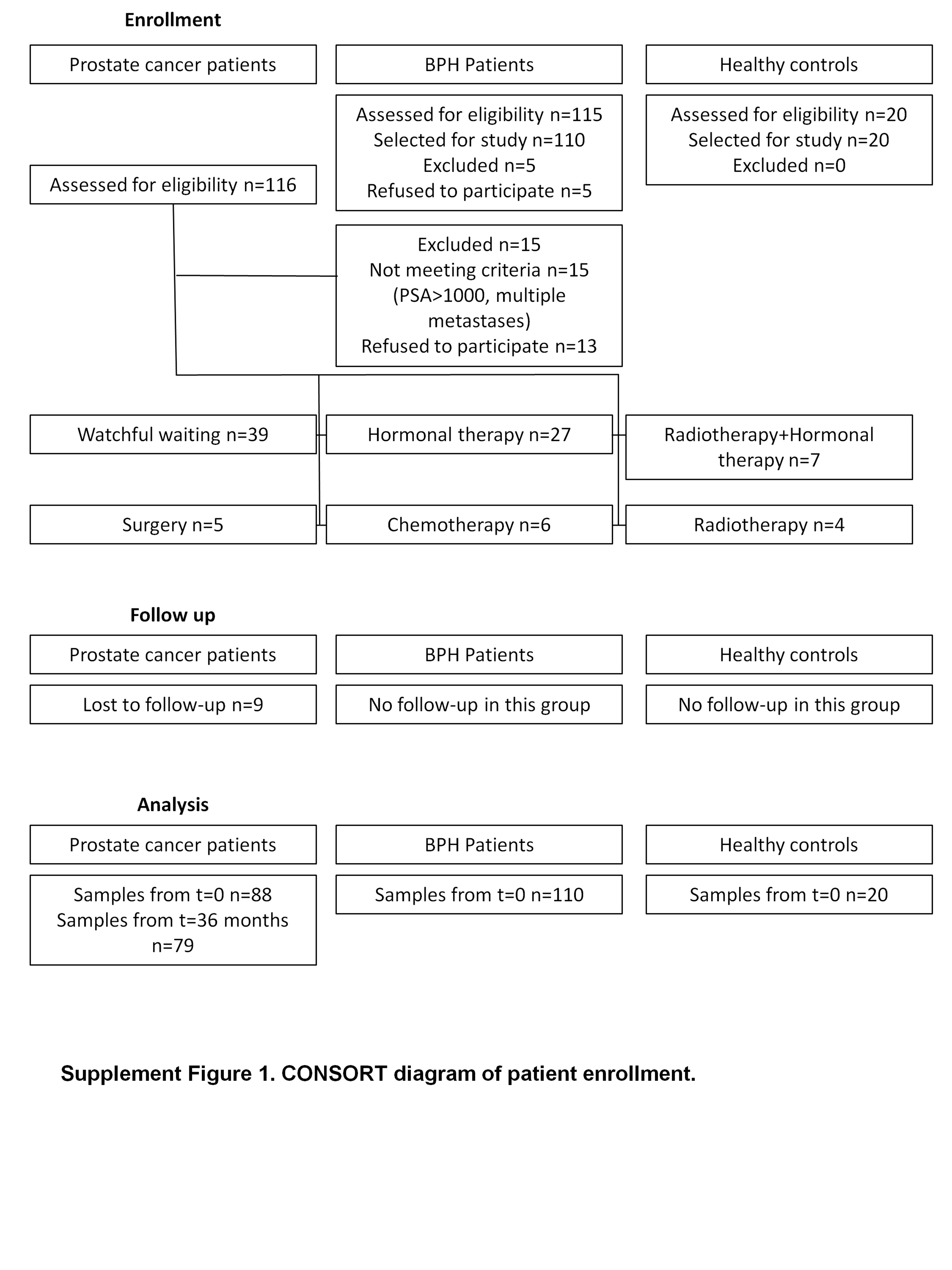

Supplement: Supplementary Figure 1 [file bjc201214x1.tif]

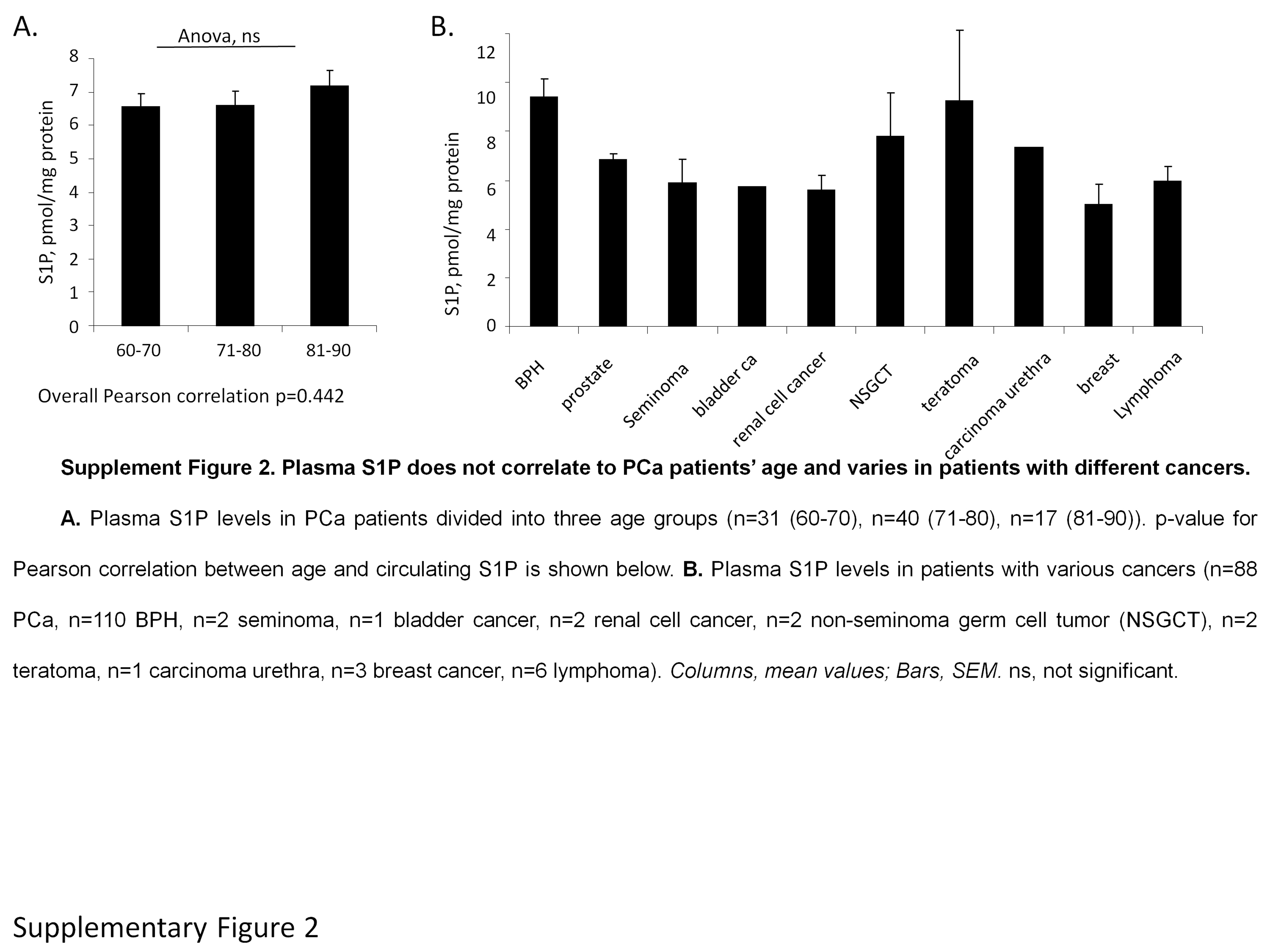

Supplement: Supplementary Figure 2 [file bjc201214x2.tif]

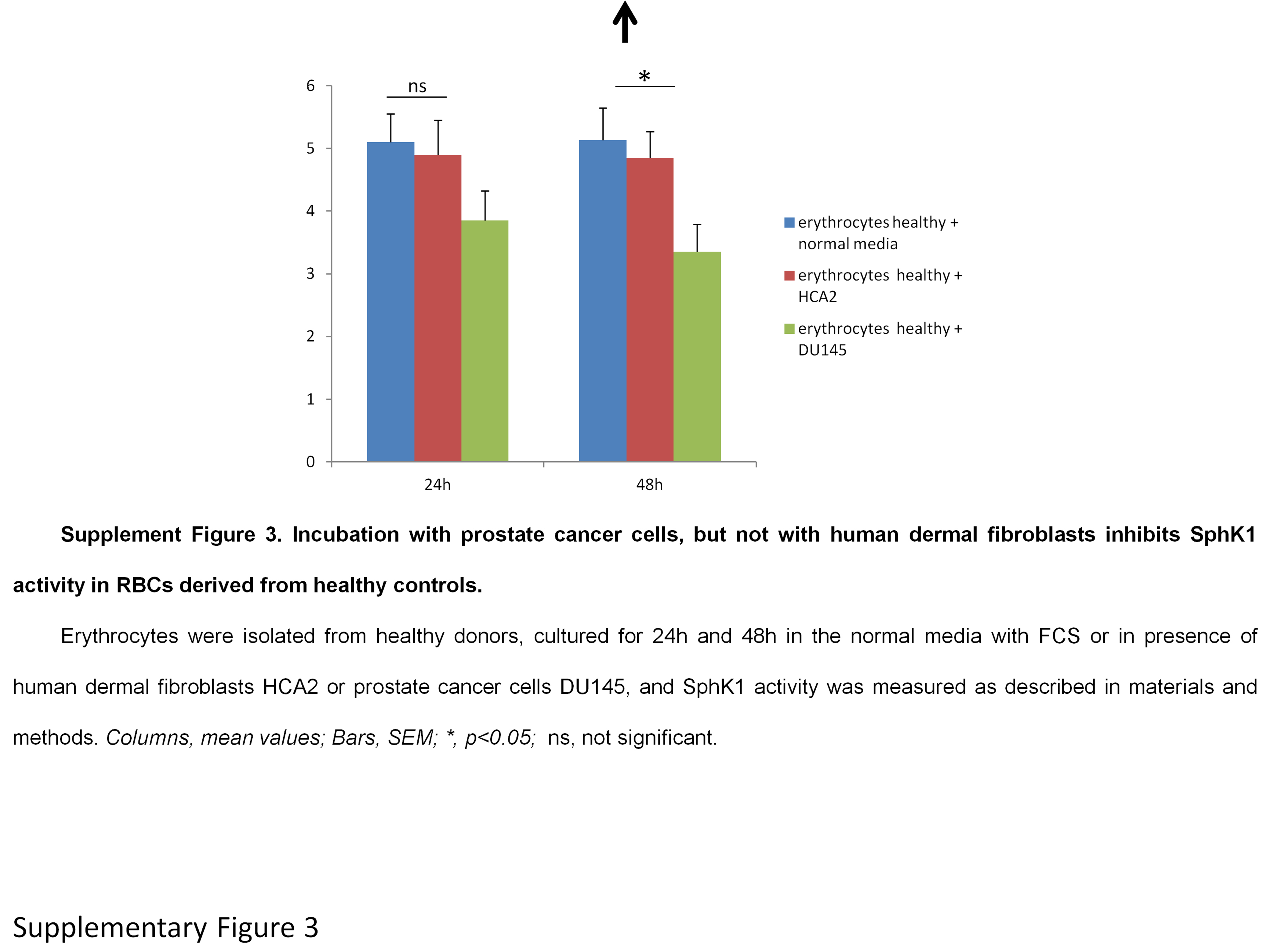

Supplement: Supplementary Figure 3 [file bjc201214x3.tif]

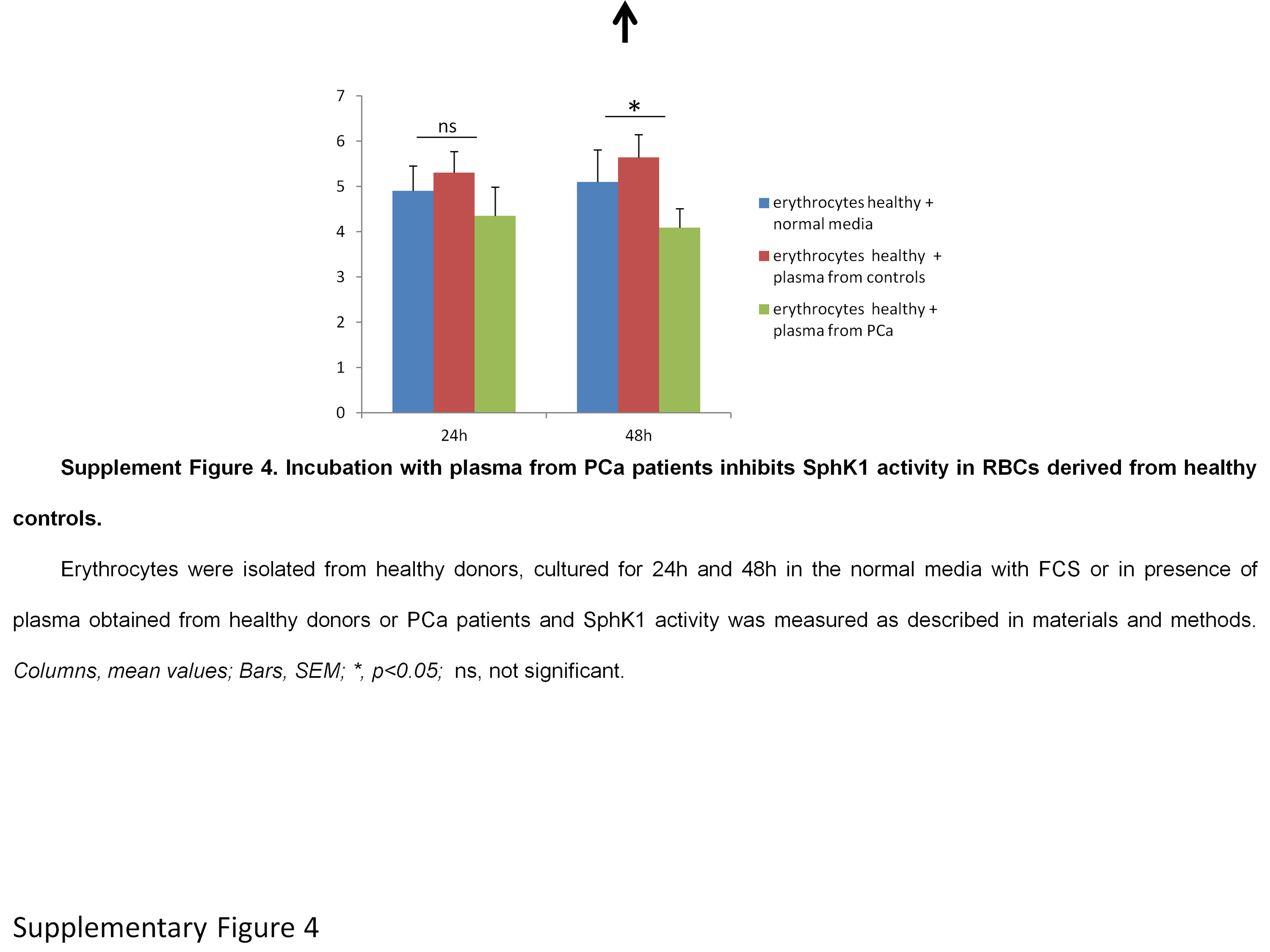

Supplement: Supplementary Figure 4 [file bjc201214x4.tif]
