# Supplementary material for: Circulating sphingosine-1-phosphate and erythrocyte sphingosine kinase-1 activity as novel biomarkers for early prostate cancer detection
Source: Br J Cancer. 2012 Feb 7;106(5):909–15. doi: 10.1038/bjc.2012.14 (PMC3305969; doi:10.1038/bjc.2012.14)
Supplement: Supplementary Information [file bjc201214x5.doc]

Supplementary table 1. Clinical characteristics of recruited PCa patients.

| **Tumor grade** | T1 | T2 | T3 | T4 |  | |
| --- | --- | --- | --- | --- | --- | --- |
| **Number of patients** | 19 | 31 | 21 | 17 |  |  |
|  | | | | | | |
| **Treatment** | None | Hormonal therapy | Radiotherapy + hormonal therapy | Surgery | Chemotherapy | Radiotherapy |
| **Number of patients** | 39 | 27 | 7 | 5 | 6 | 4 |
|  | | | | | | |
| **Metastases** |  |  | **Lymph nodes** |  |  | |
| positive | 16 |  | positive | 15 |  |  |
| negative | 72 |  | negative | 73 |  |  |

**N**=88; **Age**, mean (range): 72.8±7.8 (56-89); **PSA at recruitment** (g/L), mean (range): 50.8±76 (0.01-872); **Gleason score**, mean (range): 6.8±1.1 (5-9)

**Supplementary Table 2: Correlation between plasma S1P levels and blood cell counts.**

|  | **RBCs** | **WBCs (PBMCs)** | **Platelets** | **hGB** |
| --- | --- | --- | --- | --- |
| **S1P** | NS  p=0.1533 | *  p=0.0483 | NS  p=0.0965 | NS  p=0.2340 |

Pearson correlation p values are shown. NS - non-significant, * - p<0.05

**Supplementary Table 3. The correlation between blood cell SphK1 activity and circulating S1P in healthy controls and PCa patients.**

|  | **Healthy** | **PCa** |
| --- | --- | --- |
| **RBC count** | 5.4±0.3 | 4.5±0.4 |
| **RBCs SphK1 activity vs plasma S1P** | r=0.586  p=0.031 | r=0.416  p=0.043 |

Pearson correlation coefficient (r) and p values are shown.

**Supplementary Table 4. Changes in plasma S1P levels induced by PCa therapies.**

|  | **S1P, pmol/mg protein** | **Number of patients** |
| --- | --- | --- |
| **Watchful waiting** | 6.92 | 39 |
| **Chemotherapy** | 5.41 | 6 |
| **Radiotherapy** | 6.77 | 4 |
| **Hormonal** | 6.03 | 27 |
| **Radio+Hormonal** | 7.94 | 7 |
| **Surgery** | 8.31 | 5 |
| **Overall** | 6.88 | 88 |
|  | ANOVA p=0.079 |  |

**Supplementary Table 5. The correlation between blood cell counts and hemoglobin and PCa stage, Gleason Sum and PSA.**

|  | **stage** | **Gleason sum** | **PSA** |
| --- | --- | --- | --- |
| **WBCs (PBMCs)** | p=0.0880 | p=0.3420 | p=0.7975 |
| **RBCs** | p=0.0776 | p=0.2046 | p=0.0765 |
| **Platelets** | p=0.7043 | p=0.5523 | p=0.4342 |
| **HgB** | p=0.1453 | p=0.2393 | p=0.0872 |

Pearson correlation p values are shown.
